# Activation of ATR-Chk1 pathway facilitates EBV-mediated transformation of primary tonsillar B-cells

**DOI:** 10.18632/oncotarget.14120

**Published:** 2016-12-23

**Authors:** Vanessa Mordasini, Seigo Ueda, Roberta Aslandogmus, Christoph Berger, Claudine Gysin, Daniela Hühn, Alessandro A Sartori, Michele Bernasconi, David Nadal

**Affiliations:** ^1^ Experimental Infectious Diseases and Cancer Research, University Children's Hospital of Zürich, Zürich, Switzerland; ^2^ Department of Otolaryngology-Head & Neck Surgery, Asahikawa Medical University, Asahikawa, Japan; ^3^ Division of Infectious Diseases and Hospital Epidemiology, University Children's Hospital of Zürich, Zürich, Switzerland; ^4^ Division of Otolaryngology, University Children's Hospital of Zürich, Zürich, Switzerland; ^5^ Institute of Molecular Cancer Research, University of Zürich, Zürich, Switzerland

**Keywords:** EBV, DDR, ATR, Chk1, hyperproliferation

## Abstract

Primary infection of the immunocompromised host with the oncovirus Epstein-Barr virus (EBV) that targets mainly B-cells is associated with an increased risk for EBV-associated tumors. The early events subsequent to primary infection with potential for B-cell transformation are poorly studied. Here, we modeled *in vitro* the primary infection by using B-cells isolated from tonsils, the portal of entry of EBV, since species specificity of EBV hampers modeling in experimental animals. Increasing evidence indicates that the host DNA damage response (DDR) can influence and be influenced by EBV infection. Thus, we inoculated tonsillar B-cells (TBCs) with EBV-B95.8 and investigated cell proliferation and the DDR during the first 96 hours thereafter. We identified for the first time that EBV infection of TBCs induces a period of hyperproliferation 48-96 hours post infection characterized by the activation of ataxia telangiectasia and Rad3-releated (ATR) and checkpoint kinase-1 (Chk1). Whereas inhibition of Chk1 did not affect B-cell transformation, the specific inhibition of ATR robustly decreased the transformation efficiency of EBV. Our results suggest that activation of ATR is key for EBV-induced B-cell transformation. Thus, targeting the interaction between ATR/Chk1 and EBV could offer new options for the treatment of EBV-associated malignancies.

## INTRODUCTION

Environmental and endogenous challenges constantly endanger the integrity of the genetic information by inducing DNA damage. Eukaryotic cells have developed several powerful mechanisms to repair DNA damage, thereby preventing genomic instability and avoiding tumorigenesis [1]. Depending on the type of lesion, specific DNA damage response (DDR) protein kinases are activated. In general, single-stranded regions of DNA coated by the replication protein A (RPA) promote activation of ataxia telangiectasia and Rad3-related (ATR) [2, 3], whereas DNA double-strand breaks (DSBs) recognized by the MRE11-RAD50-NBS1 (MRN) complex primarily activate ataxia telangiectasia mutated (ATM) [4, 5]. Downstream of ATM and ATR, phosphorylation events are initiated to activate a huge variety of transducer and effector proteins, such as the serine-threonine checkpoint effector kinases Chk1 and Chk2 [6]. The latter will, in turn, phosphorylate additional effectors proteins, which can induce activation of specific DNA damage checkpoints leading to cell cycle arrest, thereby allowing time for the repair of damaged DNA. This prevents DNA replication or cell division in the presence of damaged genomic material and the spreading of possible deleterious mutations. Should DNA repair not be possible to prevent a catastrophic level of genomic instability, the DDR can activate different downstream pathways that result into programmed cell death or cell senescence and eliminate the potential threats.

There is increasing evidence that the oncogenic gammaherpesvirus Epstein-Barr virus (EBV), which mainly targets B-cells and establishes chronic B lymphocellular infection, modulates the activity of the cellular DDR [7–10]. In particular, Nikitin and colleagues showed that activation of the ATM/Chk2 signaling pathway after *in vitro* EBV infection of B-cells is critical for the suppression of EBV-mediated B-cell transformation and can act as an innate tumor suppression pathway [11].

EBV infects more than 95% of the world's population [12]. The nasopharyngeal lymphoid system, including tonsils, is the portal of entry for EBV that targets and resides in B-cells for the life-time of the host. Thus, following EBV exposure, tonsillar B-cells (TBCs) are most likely the first B-cells targeted by the virus. After primary infection, EBV establishes reversible latency in B-cells and persists there mostly as a long lasting asymptomatic infection in a rather stable pool of resting memory B-cells that circulate in the peripheral blood [13, 14]. Lytic reactivation in the nasopharynx allows host-to-host transmission of EBV via saliva to susceptible hosts [15]. Although EBV infection is harmless in the vast majority of cases, latent EBV infection is strongly associated with tumors such as endemic Burkitt's lymphoma, Hodgkin lymphoma, and post-transplant lymphoproliferative disease (PTLD) [16]. Indeed, *in vitro* infection of B-cells with EBV results in expression of all EBV's latency genes and eventually in cell transformation with the outgrowth of lymphoblastoid cell lines, thus reflecting EBV's oncogenic potential [17–19].

Primary EBV infection induces both a humoral and a cell-mediated immune response [20]. The humoral response mainly limits the spreading of the infectious virus particles blocking their binding to the cellular surface receptors [20, 21]. Cytotoxic T lymphocytes (CTL)s target and kill EBV-infected B-cells, thereby playing a key role in limiting their propagation. Immunocompromised individuals lacking a fully functional immune response, such as HIV-infected patients or organ transplant recipients, are at high risk of developing EBV-related B-cell lymphoma. Even so, the iatrogenic immunosuppression necessary to avoid graft rejection in solid organ transplantation leads to PTLD development in only up to 10% of the patients [22], suggesting that in addition to the adaptive cellular immune responses other mechanisms may play an important role in preventing the development of EBV-associated B-cell malignancies. One such additional protective mechanism could be the nature of the activated DDR since it has been identified as a major component of the underlying tumor suppressor mechanism upon EBV infection [11].

Here, we investigated the DDR in TBCs in response to EBV inoculation. We chose TBCs since they are likely the first host B-cells to be confronted with the virus upon primary infection with EBV which, in turn, is associated with the highest risk for PTLD in transplant recipients [13].

## RESULTS

### Tonsillar B-cells hyperproliferate in the first 96 hours post EBV inoculation

**Figure 1:**
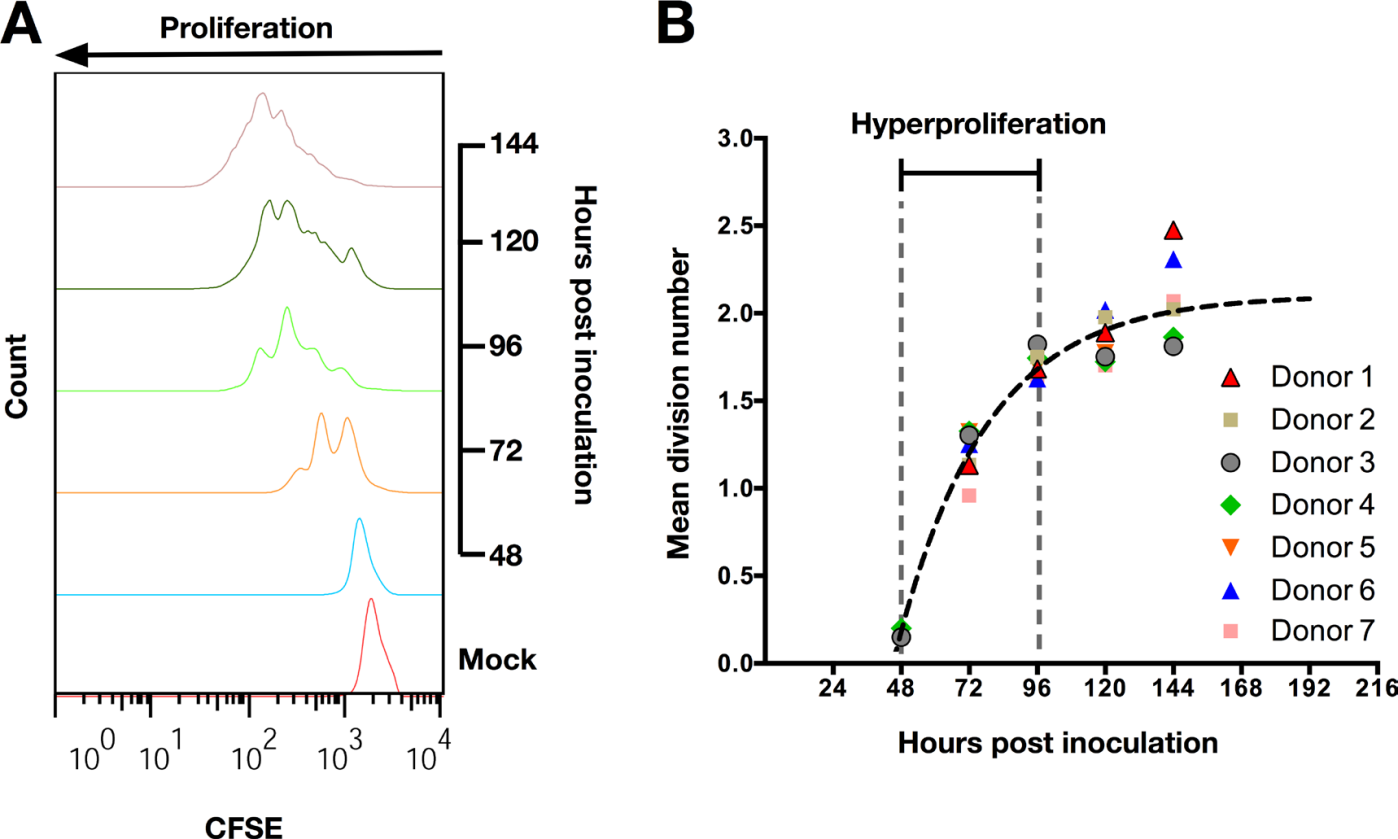
EBV induces hyperproliferation of tonsillar B-cells (TBCs) within 72 hours post inoculation (**A**) Representative data (Donor 7 from Figure 1B) of CFSE proliferation profile of TBCs at sequential days post EBV-B95.8 inoculation. Non-inoculated CD19+ B-cells harvested 120 hours post isolation (Mock) were used as negative control. (**B**) Mean division number of TBCs at sequential days post EBV-B95.8 inoculation. The function depicted as a black dashed line represents the average mean division number (MDN) for each time point measured. . Results shown are from TBCs from 7 donors.

Peripheral blood B-cells inoculated with EBV *in vitro* manifest subsequently a phase of hyperproliferation of 96 hours [11]. Since palatine tonsils are located at the portal of entry for EBV, TBCs are most likely the first B-cells to be targeted by EBV following primary infection of the host, i.e., in the absence of adaptive specific immunity. Given that TBCs and B-cells circulating in the peripheral blood may phenotypically and functionally differ [23], we interrogated whether EBV inoculation *in vitro* also induces hyperproliferation of isolated TBCs. To this end, we inoculated purified CD19+ TBCs with EBV-B95.8, produced in the marmoset B95.8 cell line exposed to 12-O-tetradecanoylphorbol-13-acetate (TPA), at a multiplicity of infection (MOI) of 8, and stained the TBCs with the proliferation dye CFSE. We monitored the proliferation of TBCs at 48, 72, 96, 120, and 144 hours post inoculation (pi) using flow cytometry (Figure 1A). Non-inoculated purified CD19+ TBCs were grown for 120 hours and used as negative control (mock inoculation). EBV-inoculated CD19+ TBCs started to proliferate after 48 hours and divided more than once between 48 and 72 hours, as indicated by the number of peaks detected by CFSE staining. In addition, EBV-inoculated CD19+ TBCs proliferated faster between 48 and 96 hours than at later time points as indicated by a rapid decrease in CFSE staining (Figure 1A). In contrast, CD19+ TBCs did not proliferate within the first 120 hours post mock-inoculation. Thus, EBV induces proliferation of CD19+ TBCs *in vitro* that peaks between 48 and 96 hours post inoculation.

Next, we wanted to investigate whether proliferating TBCs undergo a phase of hyperproliferation similar to the one reported for peripheral blood B-cells [11]. Thus, we further analyzed data from the CFSE staining experiments (Figure 1A) as suggested by Hawkins *et al*. [24]. Briefly, we calculated the mean division number (MDN) fitting a Gaussian distribution to the precursor-normalized number of cells in each division. For each TBC donor, we plotted the MDN *versus* the analyzed time point (Figure 1B). The slope of this function represents the number of divisions per hour and inversely correlates with the proliferation rate. The proliferation rate of TBCs decreased over time (Figure 1B), indicating that cells hyperproliferated between 48 and 96 hours after EBV inoculation but not thereafter.

Taken together, these data demonstrate that CD19+ TBCs behave similarly to circulating peripheral blood B-cells, undergoing a phase of hyperproliferation during the first 4 days (96 hours) post *in vitro* EBV inoculation.

### EBV induces activation of the ATR/Chk1 pathway in tonsillar B-cells

Cellular hyperproliferation implicates augmented frequency of DNA replication, which can lead to high levels of DNA damage resulting in so called replication stress [25]. ATR and its downstream effector kinase Chk1 are both key players in the cellular response to DNA replication stress [26, 27].

**Figure 2:**
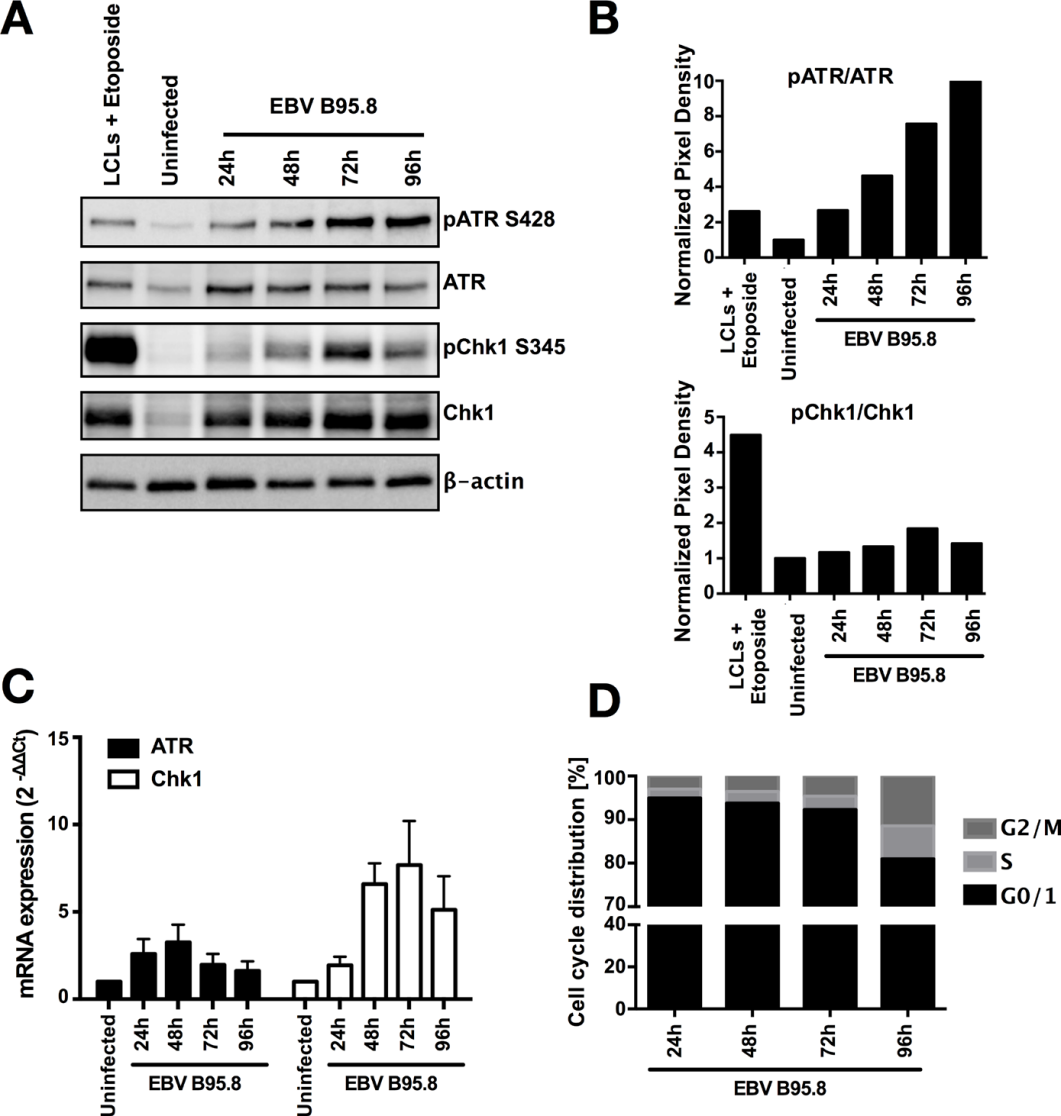
EBV inoculation elicits ATR/Chk1-mediated DDR activation in tonsillar B-cells (TBCs) TBCs where inoculated with EBV-B95.8 (MOI 8). (**A**) Cells were harvested at the indicated time points and expression of total ATR and Chk1 and pATR S428 or pChk1 S345 was analyzed by western blotting. Non-inoculated TBCs at 24 hours post isolation were used as negative control. Lymphoblastoid cell lines (LCLs) treated with Etoposide for 6 hours served as positive control. The results shown are representative of 3 independent experiments (**B**) ATR and Chk1 phosphorylation was quantified by densitometric analysis. Data are represented as ratio of phosphorylated-to-total. Quantification was performed using the software imageJ 1.49t. The results shown are representative of 3 independent experiments (**C**) ATR and Chk1 mRNA expression was determined in EBV-inoculated B-cells at the indicated time points by RTqPCR relative to 18S RNA and shown as fold change relative to non-inoculated cells. Data are presented as mean ± SEM of 4 donors. (**D**) Cell population in G1, S, G2/M at different time points post EBV-inoculation were quantified by flow cytometry based cell cycle analysis measured by labeling the fixed cells with propidium iodide (PI). Results shown are from 3 donors and are presented as mean.

To investigate if ATR and Chk1, as to be expected during hyperproliferation, are activated in TBCs in the observed hyperproliferation phase following EBV inoculation, we measured their protein expression and phosphorylation levels in purified CD19+ TBCs at 24, 48, 72, and 96 hours post EBV inoculation by western blotting (Figure 2A). As positive and negative controls, we used EBV-induced lymphoblastoid cell lines (LCLs) treated with etoposide, a DNA topoisomerase II inhibitor known to induce DSBs and SSBs and mock-inoculated purified CD19+ TBCs harvested 24 hours post-isolation, respectively. We detected a gradual increase in the phosphorylation of ATR at serine 428 (pATR) and of Chk1 at serine 345 (pChk1) during the course post EBV inoculation, with a robust signal for pChk1 at 72 hours (Figure 2A and 2B). This period coincided with the previously observed hyperproliferation phase (Figure 1B), suggesting that indeed EBV-inoculated TBCs experience replication stress during the hyperproliferation phase observed between 48 to 96 hours post EBV inoculation. Remarkably, we also observed a strong increase in total ATR and Chk1 protein levels already at 24 hours after EBV inoculation (Figure 2A). Increased ATR and Chk1 protein levels can be triggered either by increased *de novo* transcription and translation of the protein, by decreased protein degradation or by increased protein stability [28, 29]. Furthermore, analysis of both ATR and Chk1 mRNA expression levels by real-time quantitative PCR (Figure 2C) showed an increased mRNA expression for both ATR and Chk1 starting already at 24 hours post EBV inoculation. This suggested that the increase in total ATR and Chk1 protein levels was rather due to increased *de novo* transcription and translation rather than decreased protein degradation.

Kaneko *et al*. [29] showed that expression of Chk1 is increased specifically at the S to M phase of the cell cycle at both the RNA and protein levels, whereas ATR seems to be expressed throughout the cell cycle at constant levels [30]. Indeed, in our analysis of the cell cycle of EBV inoculated TBCs by PI staining we found an increase in the S-G2/M population starting at 72 hours post EBV inoculation (Figure 2D and Supplementary Figure S1A). This explained, at least in part, why we documented such a strong increase in Chk1 expression.

Collectively, the observed chronological correlation between hyperproliferation and activation of the ATR/Chk1 pathway together with an accumulation of cells in S-G2/M phase further strengthens the notion that TBCs experience a phase of replication stress early after EBV inoculation in this system with the absence of specific adaptive immunity.

### EBV does not induce activation of the ATM/Chk2 pathway in tonsillar B-cells

**Figure 3:**
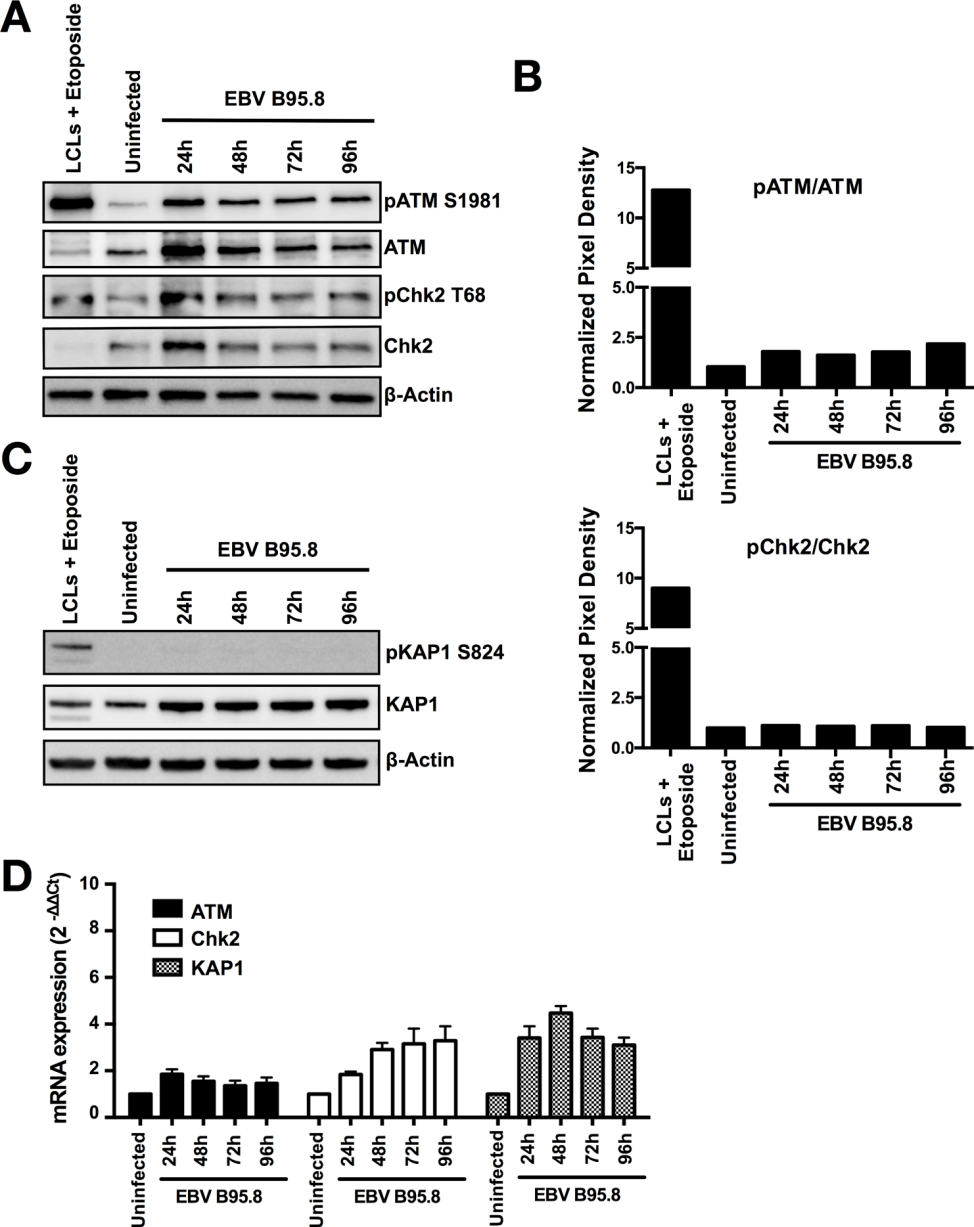
EBV inoculation does not elicit ATM/Chk2-mediated DDR activation in tonsillar B-cells (TBCs) TBCs where inoculated with EBV-B95.8 (MOI 8). Non-inoculated TBCs at 24 hours post isolation were used as negative control (uninfected). Lymphoblastoid cell lines (LCLs) treated with Etoposide for 6 hours served as positive control. (**A**) Cells were harvested at the indicated time points, and expression of total ATM or Chk2 and pATM S1981 or pChk2 T68 was analyzed by western blotting. (**B**) ATM and Chk2 phosphorylation and total protein amount were quantified by densitometric analysis. Data are represented as ratio of phosphorylated-to-total. Quantification was performed using the software imageJ 1.49t. (**C**) Total KAP1 and pKAP1 S824 was analyzed by western blotting. The results shown are representative of 3 independent experiments. (**D**) ATM, Chk2 and KAP1 mRNA expression was determined in EBV-inoculated B-cells at the indicated time points by RTqPCR relative to 18 S RNA and shown as fold change relative to non-inoculated cells. Data are presented as mean ± SEM of 4 donors.

Besides ATR/Chk1, a second kinase signaling cascade consists of the ATM and Chk2 protein kinases, which are mainly activated by DSBs rather than replication stress [31]. Furthermore, activation of Chk2 has been reported to occur following EBV infection of peripheral blood B-cells *in vitro* [11]. Therefore, we analyzed activation of ATM and Chk2 in the identical samples shown in Figure 2. Similar to ATR and Chk1, we also noted a robust increase in total ATM and Chk2 protein levels 24 hours after EBV inoculation (Figure 3A), confirmed by analysis of both ATM and Chk2 mRNA expression levels by real-time quantitative PCR (Figure 3D). In addition, we also detected a low activation of pATM and pCHk2 especially 24 hours post EBV inoculation (Figure 3A). However, the activation was limited compared to the etoposide control (Figure 3B) and did not affect activation of the ATM substrate KAP1 (pKAP1 at serine 824) that controls DSB repair in heterochromatin [32] (Figure 3C). Interestingly, similarly to ATM and Chk2, we could also observe an increase of both total KAP1 protein levels and mRNA expression levels (Figure 3C and 3D) post EBV inoculation. The absence of KAP1 activation strongly supports the absence of ATM/CHk2 pathway activation in TBCs following EBV inoculation. Nevertheless, the observed limited activation of ATM and Chk2 could explain previous reports on the activation of the ATM pathway by EBV.

Taken together, these results document that the ATM/Chk2 pathway is not activated in purified CD19+ TBCs inoculated with EBV *in vitro*.

### EBV activates the ATR/Chk1 pathway also in peripheral blood B-cells

**Figure 4:**
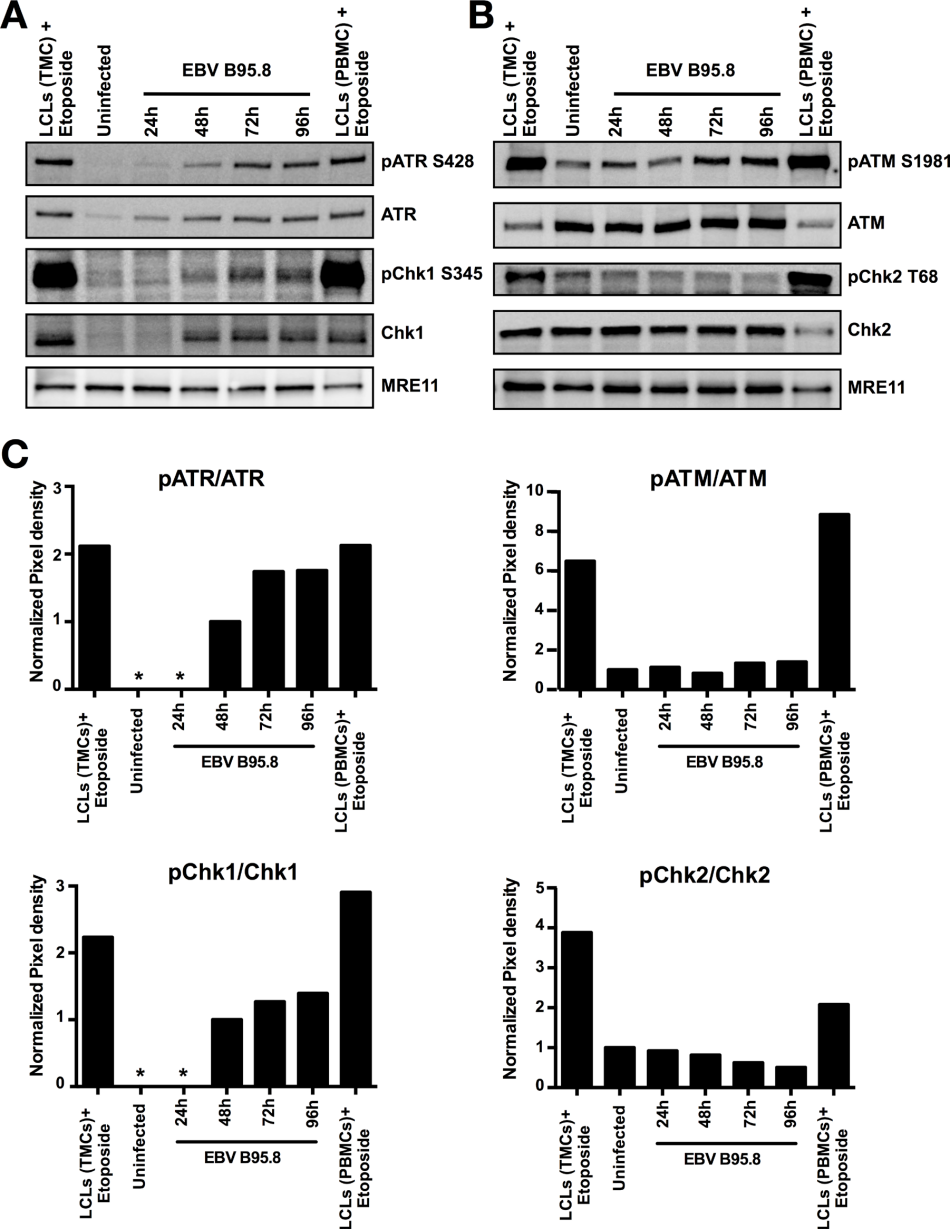
EBV inoculation elicits ATR/Chk1-mediated pathway activation but not ATM/Chk2 in peripheral B-cells (PBCs) Peripheral B-cells were inoculated with EBV-B95.8 (MOI 8). Non-inoculated peripheral B-cells at 24 hours post isolation served as negative control (Uninfected), Lymphoblastoid cell lines (LCLs) derived from TMCs or PBMCs treated with Etoposide for 1 hours served as positive control. Detection of MRE11 served as loading control. (**A**) Cells were harvested at the indicated time points and expression of total ATR or Chk1 and pATR S428 or pChk1 S345 was analyzed by western blotting. (**B**) Cells were harvested at the indicated time points, and expression of total ATM or Chk2 and pATM S1981 or pChk2 T68 was analyzed by western blotting. The results shown are representative of 3 independent experiments. (**C**) ATR, ATM, Chk1 and Chk2 phosphorylation and total protein amount were quantified by densitometric analysis. Data are represented as ratio of phosphorylated-to-total. Quantification was performed using the software imageJ 1.49t. * = Not detectable.

Given that EBV infection of peripheral blood B-cells *in vitro* was reported to activate ATM and Chk2 but the activation of ATR and Chk1 was not referred to [11], we interrogated whether activation of the ATR/Chk1 dependent pathway is dependent or independent of the infected B-cell origin, i.e., tonsil versus peripheral blood. Thus, we purified CD19+ peripheral B-cells and inoculated them with EBV-B95.8 similarly as isolated CD19+ TBCs. We then monitored the protein expression and phosphorylation levels of ATR, Chk1, ATM and Chk2 at 24, 48, 72, and 96 hours post EBV inoculation by western blotting (Figure 4A and 4B). Similarly to TBCs (Figures 2A and 3A), we observed activation of the ATR/Chk1 dependent pathway in peripheral blood B-cells following EBV inoculation, indicated by a reproducible increase of the phosphorylation for both ATR and Chk1 (Figure 4A and 4C). This suggested that activation of ATR and Chk1 following EBV inoculation *in vitro* is not dependent on the origin of the B-cells.

Surprisingly, in contrast to what was reported we did not detect activation of the ATM/Chk2 dependent pathway in peripheral blood B-cells following EBV inoculation (Figure 4B and 4C) except for a minimal increase of the ATM total-to-phosphorylation ratio after EBV infection similar to what we observed in TBCs

(Figure 3B). This suggested that distinct experimental procedures in the reported study [11] versus our study were likely responsible for the contrasting results in terms of ATM/Chk2 activation in peripheral blood B-cells.

### Activation of ATR/Chk1 is not EBV-strain dependent

**Figure 5:**
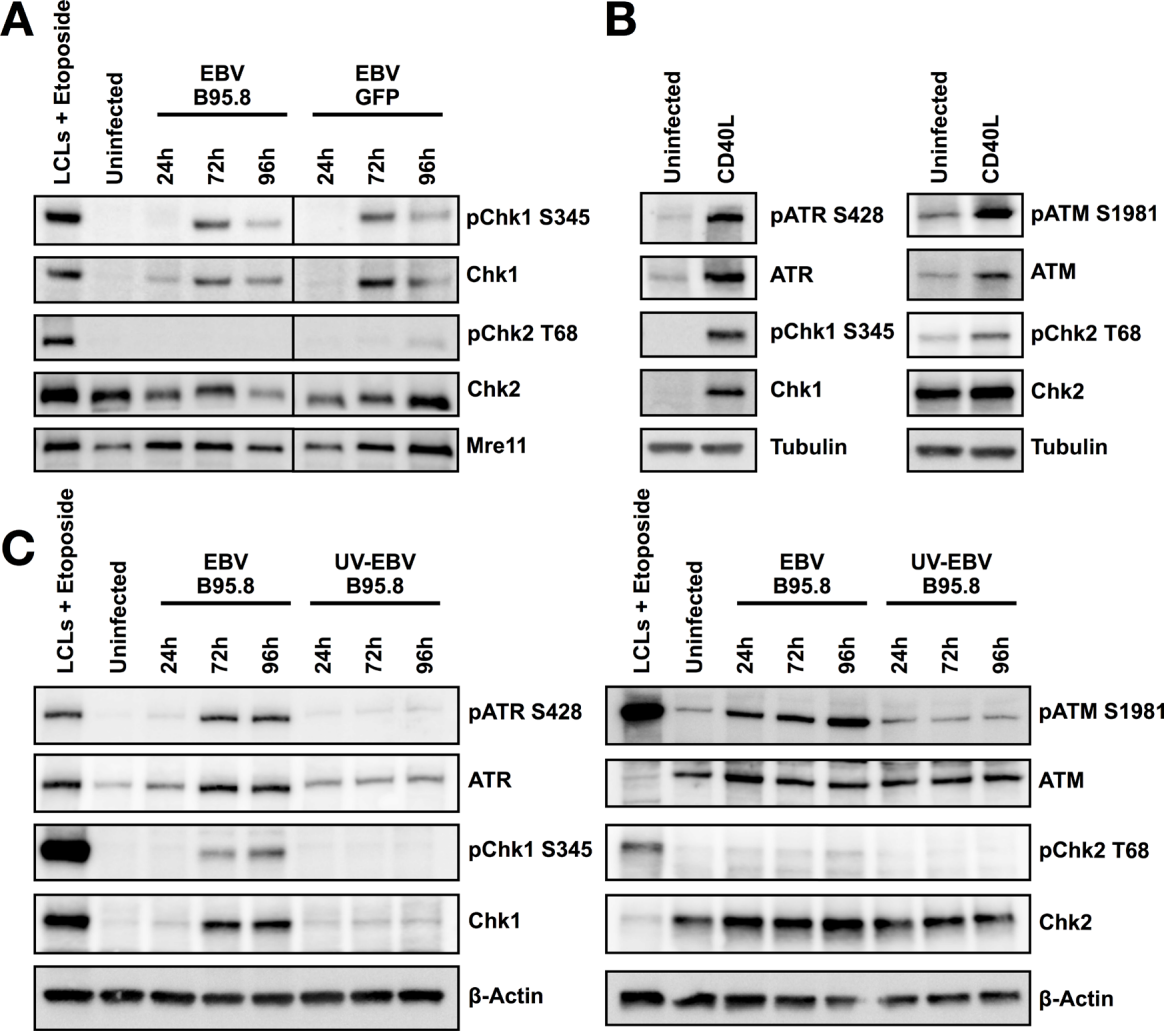
Hyperproliferation of CD19+ TBCs induced by CD40L activates Chk1 but not inoculation with UV inactivated EBV-B95.8 activates Chk1 (**A**) TBCs were inoculated with EBV-B95.8 or EBV-GFP (MOI 8). Cells were harvested at the indicated time points and expression of total Chk1 and pChk1 S345 or total Chk2 and pChk2 T68 was analyzed by western blot. Non-inoculated TBCs at 24 hours post isolation served as negative control, lymphoblastoid cell lines (LCLs) treated with Etoposide for 1 hour served as positive control. Detection of MRE11 served as loading control. The results shown are representative of 3 independent experiments. (**B**) TBCs where treated with 5 ng/mL CD40L, 20 ng/mL Il-4 and 200 ng/mL HA-antibody for 72 h. Expression of pATR, pChk1, pATM or pChk2 was analyzed by western blot. Non-inoculated TBCs at 24 hours post isolation served as negative control. Tubulin served as loading control (**C**) TBCs were inoculated with EBV-B95.8 or UV-EBV-B95.8 (MOI 8). Cells were harvested at the indicated time points and expression of pATR, pChk1, pATM or pChk2 was analyzed by western blotting. Non-inoculated TBCs at 24 hours post isolation served as negative control, lymphoblastoid cell lines (LCLs) treated with Etoposide for 6 hours served as positive control. Detection of ß-Actin served as loading control. The results shown are representative of 3 independent experiments.

Next, we interrogated whether activation of the ATR/Chk1 signaling pathway in CD19+ TBCs is dependent on the strain of EBV used for inoculation. To this end, we monitored Chk1 and Chk2 phosphorylation following inoculation of TBCs with two distinct EBV strains. We inoculated purified CD19+ TBCs with EBV-B95.8 or with EBV-GFP produced in HEK-293 cells. Consistent with our previous results, we observed that pChk1, but not pChk2, was induced at 72 hours post-inoculation either with EBV-B95.8 or with EBV-GFP (Figure 5A).

Thus, activation of the ATR/Chk1 pathway in TBCs following inoculation is not an EBV strain-dependent phenomenon, at least not with the two strains tested.

### Hyperproliferation of CD19^+^ TBCs induced by CD40L activates Chk1

Since ATR/Chk1 activation does not seem to be EBV-strain specific, we asked whether the observed activation of ATR/Chk1 was EBV-dependent at all. Thus, we wondered whether CD40L, a B-cell stimulant with similar effects as EBV regarding B-cell survival and hyperproliferation [33], did also activate ATR/Chk1. To answer this question, we used CD40L in combination with IL-4 to stimulate proliferation of TBCs similar to the one observed in EBV-infected B-cells but in an EBV-independent fashion. We treated purified CD19+ TBCs with CD40L in combination with IL-4 for 72 hours and measured the levels of, pATR, pChk1, pATM and pChk2 by western blotting. Indeed, inoculation of isolated CD19+ TBCs with CD40L resulted in activation of ATR and Chk1 at 72 hours post stimulation (Figure 5B).

Thus, considering that peripheral blood B-cells undergo a phase of hyperproliferation at 72 hours post CD40L stimulation [33], activation of the ATR/Chk1-dependent pathway seems not to be a strictly EBV-related phenomenon in B-cells but rather seems to be linked to the hyperproliferation of the B-cells.

To verify if activation of the ATR/Chk1 pathway is dependent from productive EBV infection or rather form EBV or cellular components that might have co-purified with the virus, we monitored ATR, Chk1, ATM and Chk2 following inoculation of purified CD19+ TBCs with EBV-B95.8 or UV-inactivated EBV-B95.8. Contrasting with ATR/Chk1 activation following inoculation with live EBV-B95.8, UV-inactivated EBV-B95.8 did not show any phosphorylation of ATR and Chk1 (Figure 5C). This demonstrated that the UV-inactivated EBV does not activate ATR/Chk1-dependent pathway as live EBV, excluding that other elements present in the viral supernatant are responsible for Chk1 activation. Thus, TBCs need to experience EBV infection rather than mere contact with EBV or its elements to manifest ATR/Chk1 activation during hyperproliferation following EBV inoculation (Figure 1A).

Taken together, these results confirm that hyperproliferation is sufficient to induce activation of ATR/Chk1 in purified CD19+ B-cells This, in turn, may indicate that B-cells counteract their vulnerable phase of primary EBV infection mirrored by hyperproliferation by activating the ATR/Chk1-related pathway.

### ATR activation increases EBV-mediated transformation of TBCs

EBV has the ability to transform B-cells *in vitro* [13, 34]. Thus, we were interested to know whether activation of the specific ATR/Chk1-mediated pathway within 96 hours post EBV inoculation *in vitro* has any consequences for the subsequent transformation of TBCs, since the risk for PTLD in transplant recipients is highest following primary EBV infection but is also present in EBV carriers [22].

More specifically, we mimicked the *in vivo* situation of primary EBV B-cell infection in iatrogenically immunosuppressed transplant recipients by using tonsillar mononuclear cells (TMCs), rather than purified CD19+ TBCs, from EBV seronegative donors, to mimic primary host EBV infection. We also used TMCs from EBV seropositive donors treated with cyclosporine A to suppress T cell function, to mimic primary B-cell infection in immunosuppressed EBV carriers. We used two distinct inhibitors: VE-821, specific for ATR (ATRi) [35]; and CHIR-124, specific for Chk1 (Chk1i) [36]. Specificity of VE-821 and CHIR-124 were assessed by monitoring the phosphorylation levels of Chk1 at S345 and S296 [37] respectively (Supplementary Figure S2A and S2B). To avoid artifacts that may result from inhibitors toxicity we also assessed the cell viability after treatment with VE-821 and CHIR-124 at different concentration (Supplementary Figure S2C).

**Figure 6:**
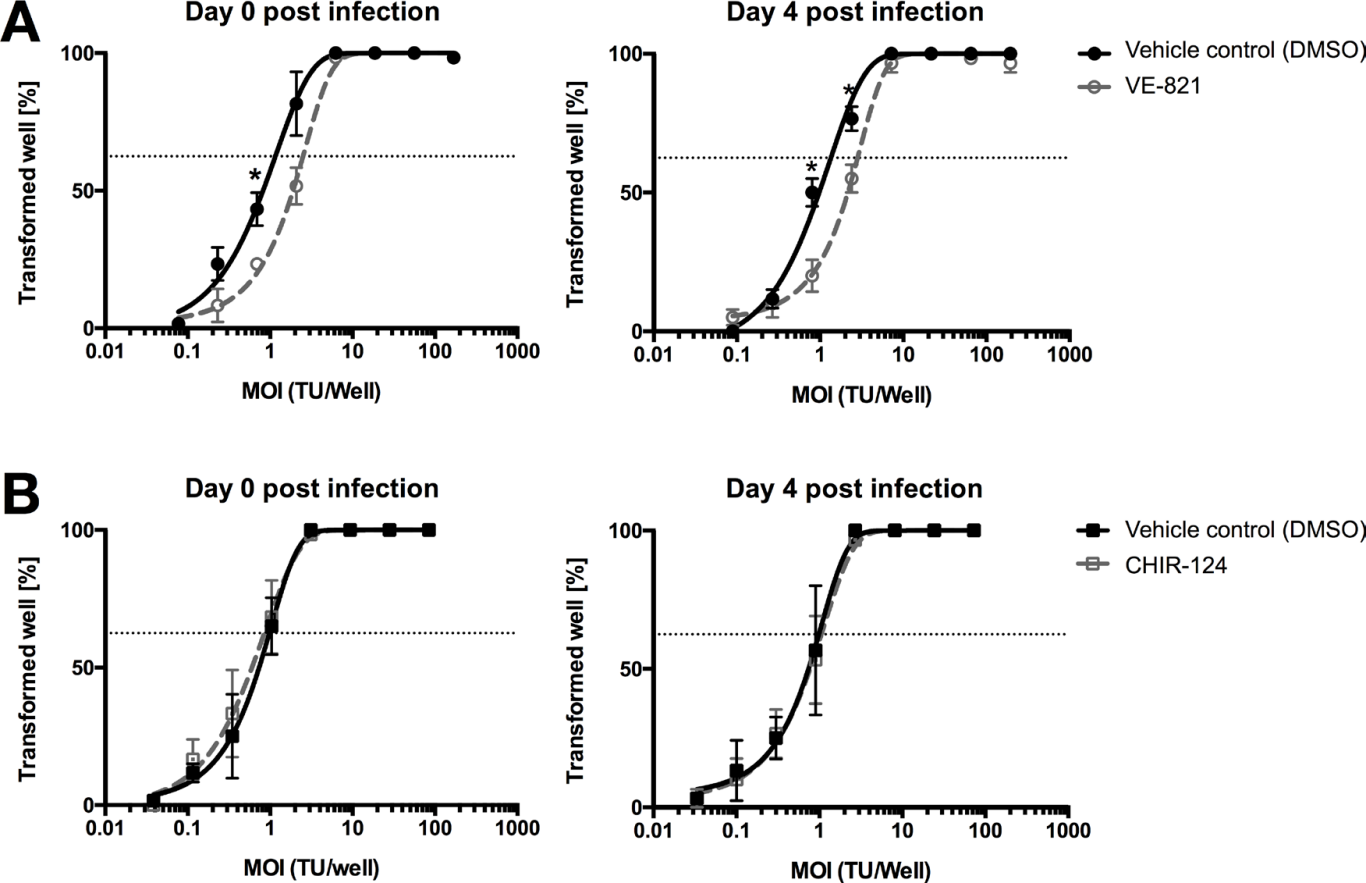
ATR inhibitor reduces EBV's B-cell transformation efficiency (**A**) Quantification of B-cells outgrowth following tonsillar mononuclear cells (TMCs) infection with EBV-95.8 (3 fold serial dilution) in presence of vehicle control (0.01% of DMSO - black line) or 1 μM VE-821 ATR inhibitor (dottet-grey line) added at day 0 or 4 post EBV inoculation. (**B**) Quantification of B-cells outgrowth following TMCs in presence of vehicle control (0.01% DMSO - black line) or 10 nM of CHIR-124 Chk1 inhibitor (dotted-grey line) added at day 0 or 4 post EBV inoculation. The number of transformed wells is plotted relative to the transforming units (TU) of EBV-B95.8 per well. Results shown are from 3 donors and are presented as mean ± SEM. For both figure (A) and (B) two EBV positive and one EBV negative donors were used. Dotted lines represent 62.5% positive wells. Based on a Poisson distribution this percentage represents the probability of B-cells outgrowth knowing that a viral particle is present in every well. *= *p* < 0.05 (Unpaired *t*-Test).

TMCs were treated with ATRi or Chk1i from the start of the inoculation (day 0 pi) or whilst in the hyperproliferation phase (4 days pi). Specifically, we inoculated TMCs with decreasing MOIs of EBV according to a 3-fold serial dilution. At 5 weeks pi, we determined the EBV transformation efficiency by counting the number of wells positive for B-cells outgrowth. The percentage of transformed wells was plotted relative to the transforming units (TU) of EBV used per well. TUs per well were calculated assuming that 62.5% of wells were transformed by 1 TU. Transformation efficiency was assessed comparing how many TUs were needed to transform 62.5% of wells. ATR inhibition at day 0 or 4 pi resulted in a decrease in transformation efficiency compared to the vehicle control DMSO of ~ 1.5 and to ~ 2.3 fold respectively (Figure 6A). By contrast, inhibition of Chk1 did not affect EBV transformation efficiency neither at day 0 pi nor 4 pi compared to the DMSO control (Figure 6B).

In conclusion, activation of ATR during the first 4 days pi, i.e. during the phase of hyperproliferation post EBV inoculation, seemed to be a key factor, which increased EBV transformation efficiency *in vitro*. Thus, factors impacting on this phase of vulnerability are likely to determine whether eventually EBV-induced transformation or PTLD occur.

## DISCUSSION

Here, we investigated the role of the DNA damage response (DDR) pathways in controlling proliferation and transformation of TBCs during EBV primary infection using an *in vitro* system to model primary EBV infection of the EBV naïve host without and with immunosuppression, the latter close to primary B-cell infection in the immunosuppressed transplant recipient. We found that following inoculation with EBV (i) TBCs undergo a phase of hyperproliferation; (ii) in that phase TBCs express pATR and pChk1, suggesting that these abnormally proliferating cells experience replication stress; and (iii) ATR inhibition in TBCs decreases EBV's transformation efficiency. Our results unprecedentedly show how a cellular mechanism, aimed at protecting DNA from damage induced by abnormal cell proliferation, opens a vulnerable phase to transformation for the target cells following primary EBV infection of the host or of a B-cell.

Remarkably, analysis of TBCs proliferation revealed that B-cells enter their first round of cellular DNA replication only between 48 to 72 hours after EBV inoculation (Figure 1A). Subsequent mathematical analysis (Figure 1B) showed that TBCs undergo a phase of hyperproliferation between 48 and 96 hours post EBV inoculation. Our observation is comparable to the findings by Nikitin *et al*. [11] who described a phase of hyperproliferation approximately between 2.5 and 4.5 days after EBV infection of peripheral blood B-cells *in vitro*. During hyperproliferation, defined as an abnormally high rate of cell division, there are higher chances for the cells to drive a faulty DNA replication. This can result in high level of replication stress and subsequently in activation of a strong and specific DDR [25]. Human Herpesvirus 6 has been shown to induce activation of Chk1 72 hours pi [38], similarly to EBV (human herpes virus 4) as documented here. Thus, activation of an ATR/Chk1-dependent pathway in EBV-inoculated TBCs, as shown here for the first time, might be an alternative mechanism of defense activated by target cells in the absence of a functional CTLs response limiting outgrowth and transformation of EBV-infected B-cells.

Of note, B-cells exhibited activation of ATR and Chk1 as indicated by phosphorylation of S428-ATR and S345-Chk1 (Figures 2A, 4A), which strictly coincided with the hyperproliferation phase following exposure to EBV. It has been shown that in response to CD40L/IL4 stimulation B-cells undergo an initial burst of hyperproliferation similar to what has been described for EBV infection [33]. Since we observed similar activation of ATR and Chk1 in TBCs following stimulation with CD40L/IL-4 (Figure 5B) the process is not EBV-specific but rather a consequence of B-cell activation leading to replication stress. Nevertheless, the absence of ATR/Chk1 activation when inoculating TBCs with UV-inactivated EBV (Figure 5C) strongly implies that EBV needs to actively infect the target cell to induce ATR/Chk1 activation.

By contrast, TBCs did not exhibit detectable activation of ATM and Chk2 in any of our experiments (Figures 3A and 4A and 5A), suggesting absence of DSB leading to the activation of the required ATM/Chk2-mediated DDR. Our results are at variance to those of Nikitin *et al*. [11] who observed activation of the ATM/Chk2-mediated DDR in peripheral blood B-cells following EBV infection *in vitro*. One possible explanation could be the distinct EBV sources used in the experiments. We exposed TBCs to a second EBV strain (EBV-GFP) and obtained the similar DDR pattern as we obtained with EBV-B95.8, suggesting that distinct EBV strains are not a very likely explanation for the results at variance. An alternative explanation could be the distinct sources of B-cells (TBCs versus peripheral blood B-cells). We executed the assessment of DDRs also with peripheral blood B-cells, but the results did not differ from those obtained with TBCs (Figure 4A). Thus, although the reason for the results at variance remains elusive, distinct experimental conditions but not the virus strains or the B-cell tissue origin are the most likely cause.

A most remarkable and surprising finding was the reduced EBV *in vitro* transformation efficiency observed after ATR inhibition (Figure 6A), indicated by the increase in the number of TUs necessary to transform 62.5% of cells after ATR inhibition. No such changes were detected after Chk1 inhibition (Figure 6B). Interestingly, Nikitin and colleagues [11] described that inhibition of ATM and Chk2 exhibited an opposite effect on EBV transformation efficiency in peripheral blood B-cells, i.e., an increased transformation efficiency of EBV. This suggested that activation of this specific DDR pathway works as an alternative tumor suppressive pathway that regulates B-cell transformation. Thus, distinct activation of specific DDRs seems to have diverse effects on EBV-induced B-cell transformation *in vitro*. This, in turn, may imply that depending on the activated DDR upon EBV exposure in the absence of adaptive immune control of EBV-infected B-cells distinct outcomes of EBV-mediated B-cell transformation may arise. Further exploration of the mechanisms involved may reveal potential interventional targets for patients with deficient immunity against EBV, e.g., organ transplant recipients or HIV-infected individuals.

Even though EBV plays a crucial role in the development of PTLD, *in vivo* only 10% of the transplant recipients develop EBV-related lymphoma. Thus, additional factors might be required in order to induce PTLD [22]. One could speculate that previous bacterial or viral infections might stimulate TBCs proliferation leading to replication stress and consequently activation of the ATR/Chk1-mediated DDR, increasing the probability of EBV transformation by creating an environment promoting lymphoblastoid cell transformation by EBV. Indeed, it is known that TLRs signaling, including TLR9 that bind specific common patterns on bacterial DNA (CpG motifs), can directly induce B-cell proliferation [33, 39]. Furthermore, it has been shown in our lab that triggering of TLR9 signaling in a Burkitt's lymphoma derived cell line blocks reactivation of EBV lytic cycle, reinforcing the risk of cell transformation [40]. Additional factors might also be required *in vivo*, in addition to ATR, to significantly increase the risk of B-cell transformation by EBV. Those factors might be expressed only by certain individuals and therefore explain why PTLD is not more prevalent within the immunosuppressed patients.

Taken together, our results indicate that activation of the specific ATR/Chk1-mediated DDR pathway, and more precisely of ATR, is important to enhance EBV transformation efficiency in TBCs *in vitro*. *In vivo*, the immunocompetent host is able to keep hyperproliferating B-cells under control and EBV causes only very rarely B-cell tumors. By contrast, transplantation-related or otherwise highly immunosuppressed patients are at high risk of developing EBV-related PTLD upon primary EBV infection. Without an efficient adaptive immune system able to restrict proliferation of EBV-infected B-cells, those latter would not be restricted to undergo hyperproliferation.

## MATERIALS AND METHODS

### Ethics statement

This study was conducted according to the principles expressed in the Declaration of Helsinki. The Cantonal Ethics Committee approved the study (StV29/06). All subjects or their caregivers provided written informed consent for the collection of samples and subsequent analyses.

### Cell culture

All cells were maintained in RPMI-1640 medium (Sigma-Aldrich, Buchs, Switzerland) supplemented with 10% heat-inactivated (hi) fetal bovine serum (FBS; Life Technologies-Thermo Fisher Scientific, Reinach, Switzerland), 2 mM L-Glutamine, and 100 U/ml penicillin, and 100 μg/ml streptomycin, referred to hereafter as complete medium. All cells were cultured at 37°C in 5% CO_2_ air of relative humidity > 95%.

### Preparation of primary B-cells

Primary human tonsillar mononuclear cells (TMCs) were isolated from palatine tonsils obtained from pediatric patients who underwent tonsillectomy due to tonsillar hyperplasia. Tonsillar B-cells (TBCs) were prepared as described previously [23]. Briefly, tonsils were cut into small pieces with a scalpel in phosphate-buffered saline (PBS) and passed through a 70 μm-pore-size cell strainer (Falcon, Wohlen, Switzerland). Then, TMCs were purified by density gradient centrifugation with Ficoll-Paque Premium (VWR international-GE Healthcare, Dietikon, Switzerland). TBCs were isolated from TMCs using the B-cell isolation kit II according to the instructions of the manufacturer (Miltenyi Biotech, Bergisch Gladbach, Germany). The purity of isolation was always over 95% as assessed by flow cytometry.

Peripheral B-cells (PBCs) were isolated from blood form healthy donors and prepared as following. Buffycoats were diluted 5× with phosphate-buffered saline (PBS). Then, peripheral blood mononuclear cells (PBMCs) were purified by density gradient centrifugation with Ficoll-Paque Premium (VWR international-GE Healthcare). PBCs were isolated from PBMCs using the B-cell isolation kit II according to the instructions of the manufacturer (Miltenyi Biotech).

### Preparation of virus stock and quantification

The EBV-infected marmoset B95-8 were seeded at a density of 10^6^ cells/ml and were stimulated to release virus by culture for 6–7 days in complete medium containing 50 ng/ml of 12-Otetradecanoylphorbol-13-acetate (TPA; Sigma-Aldrich)/ml. Cell suspensions were centrifuged at 1000 × g for 10 min. Supernatant was passed through a 0.45 μm-pore-size cellulose acetate filter (Sarstedt, Sevelen, Switzerland) and stored at −80°C.

Recombinant EBV-GFP was produced in 293 cells as described elsewhere [41]. Briefly, 80–90% confluent HEK293-D2089 [42] cultured in DMEM supplemented with 10% hiFBS, 1% L-Glutamine, 1% Penicillin/Streptomycin, 100 μg/ml Hygromycin B (HygroGOLD; InvivoGen, Toulouse, France) were transfected with expression plasmids encoding the EBV gene BZLF1, and BALF4 (2 μg each/10cm plate) using Metafectene (Biontex, Martinsried/Planegg, Germany). Four hours after transfection, the transfection mixture was replaced by fresh supplemented DMEM without Hygromycin B. Four days after transfection, supernatant was harvested, cleared by centrifugation at 4°C with 1.000 × g for 15 min, filtered through a 0.45μm filter and stored at −80°C.

Concentrated virus stocks were prepared by centrifugation of viral supernatant with 30.000 × rpm for 2 hours at 4°C and resuspension of the virus pellet in complete medium (1/100 of the starting volume) and store at −80°C.

Ultraviolet (UV)-inactivated EBV-B95.8 was prepared by exposing 1.5 ml of concentrated virus stock for 15 min to a source of UV germicidal light.

### Infection of primary B-cells and generation of lymphoblastoid cell lines (LCLs)

TBCs were centrifuged and resuspended in complete medium at a concentration of 2 × 10^6^ cells/ml. B95.8 culture supernatant prepared as described previously [23], EBV-GFP or UV-inactivated EBV-B95.8 was added to each well at an MOI of 8.

Freshly isolated TMCs or PBMCs were infected with 100 μl of B95-8 EBV and immediately treated with cyclosporin A (400 ng/ml; Sigma-Aldrich) to avoid killing of EBV-infected cells by EBV-specific T cells. The cells were then seeded on a 96-wells plate and kept in culture for at least 5 weeks before expanding and freezing them in FCS/10% DMSO at −80°C.

### Proliferation assays

TBCs were labeled with 5 μM carboxyfluorescein diacetate succinimidyl ester (CFSE; Sigma-Aldrich) for 5 min at room temperature. The cells were then washed three times with PBS containing 5% hiFBS and resuspended in complete medium. The CFSE-labeled TBCs were inoculated with B95.8 culture supernatant and kept in culture. The cells were then harvested at indicated time points and analyzed by FACSCanto II (Becton Dickinson, Allschwil, Switzerland). The percentage of divided cells was calculated using FlowJo proliferation platform and the mean division number was calculated as reported previously [24].

### Western blotting

For western blotting whole cell lysate was prepared with 1% Triton X-100 Buffer (50 mM Tris-HCL, 150 mM NaCl, 1% Triton X-100, 1 mM EGTA, 50 mM NaF 10 mM b-glycerolphosphate, 5 mM SodiumPyrophosphate, 1 mM Na_3_VO_4_) supplemented with CompleteMini protease inhibitor (Roche Diagnostics, Rotkreuz, Switzerland). For the detection of primary antibodies, we used horseradish peroxidase-labeled goat anti-rabbit or horse anti-mouse antibodies (CellSignaling, Danvers, MA). Detection was performed using ECL western blotting detection reagents (VWR international-GE Healthcare) or SuperSignal west femto kit (Thermo Fisher Scientific). The signal was detected using the imaging system Fujifilm LAS-3000 imager (Fujifilm, Dielsdorf, Switzerland). The densitometry analysis was performed using the Image J software.

### RNA extraction and quantitative real-time polymerase chain reaction (qPCR)

Total RNA was extracted using the RNeasy Mini Kit (Qiagen, Basel, Switzerland) according to the manufacturer's instructions. After DNaseI (DNA-free; Life Technologies - Thermo Fisher Scientific) treatment, 0.5 μg-1 μg of total RNA was used as template for reverse transcription using a High-Capacity cDNA Reverse Transcription Kit (Thermo Fisher Scientific). Gene expression of *Chk1*, *ATR, Chk2, ATM and KAP1* was determined using pre-validated primer/probes assay (Hs00992123_m1, Hs0967506_m1, Hs00200485_m1, Hs00175892_m1 and Hs00232212_m1 respectively; Applied Biosystems - Thermo Fisher Scientific). All reactions were performed on a 7900HT real-time PCR machine ((Applied Biosystems - Thermo Fisher Scientific) with TaqMan Gene Expression Master Mix (Thermo Fisher Scientific). The relative gene expression was calculated for each gene of interest by using a ΔΔC_t_ method, where C_t_ values were normalized to the housekeeping genes 18s RNA.

### Cell cycle analysis by flow cytometry

TBCs inoculated with B95.8 were harvested at the indicated time point and washed with PBS. The cells were then fixed in 70% ethanol and kept at 4°C until staining. After fixation the cells were washed wit PBS and stained with a propidium iodide (PI)-RNAseA solution (25 μg/ml-0.1 mg/ml) for 30 min at 37°C. Stained samples were analyzed by FACS Canto II (Becton Dickinson, Allschwil, Switzerland). The results analysis was performed using the FlowJo software.

### Cell viability assay

Viability of LCLs after VE-821 or CHIR-124 treatments was assayed using WST-1 (water soluble tetrazolium-1) salt as a substrate, as described in the protocol supplied with the kit (Roche Diagnostics, Rotkreuz, Switzerland). The absorbance measured using a microplate reader (Bio-TEK instruments, Luzern, Switzerland) at 450 nm. Cell viability was calculated by A_treatment_/A_control_ × 100 (A represents the absorbance recorded at 450 nm).

### Transformation assay

Infection of TMCs by EBV-B95.8 was performed in presence 400 ng/ml of cyclosporine A (Sigma Aldrich), 0.01% DMSO, 1μM ATRi VE-821 (Selleckchem, Huston, TX) or 10 nM Chk1i CHIR-124 (Selleckchem) added at different times post infection (pi). TMCs were plated in 24 wells of a 96 well plate and infected with different EBV concentration (MOI from 30 to 0.014, 3-fold serial dilution). The percentages of wells positive for B-cell transformation at 5 weeks post infection were plotted relative to the amount of virus used per well.

### Antibodies

Primary antibodies used in these studies included rabbit anti-pChk2 (Thr68) (clone C13C1; Cell Signaling), rabbit anti-Chk2 (Cell Signaling), rabbit anti-pATR (Ser428) (Cell Signaling), rabbit anti-ATR (Cell Signaling), rabbit anti-pChk1 (Ser345) (clone 133D3; Cell Signaling), rabbit anti β-actin (Cell Signaling), mouse anti-ATM (clone 2C1; GeneTex, Irvine, CA), rabbit anti-KAP1 (GeneTex), rabbit anti-pKAP1 (S824) (Bethyl, Montgomery, TX), rabbit anti-pATM (Ser1981) (Clone EP1890Y, Abcam, Cambridge, UK), mouse anti-Chk1 (clone G-4; Santa Cruz, Heidelberg, Germany).

## SUPPLEMENTARY MATERIALS FIGURES AND TABLES


