# Supplementary material for: Activation of ATR-Chk1 pathway facilitates EBV-mediated transformation of primary tonsillar B-cells
Source: Oncotarget. 2016 Dec 23;8(4):6461–74. doi: 10.18632/oncotarget.14120 (PMC5351645; doi:10.18632/oncotarget.14120)
Supplement: Supplementary file 1 [file oncotarget-08-6461-s001.pdf]

# Activation of ATR-Chk1 pathway facilitates EBV-mediated transformation of primary tonsillar B-cells

## Supplementary Materials

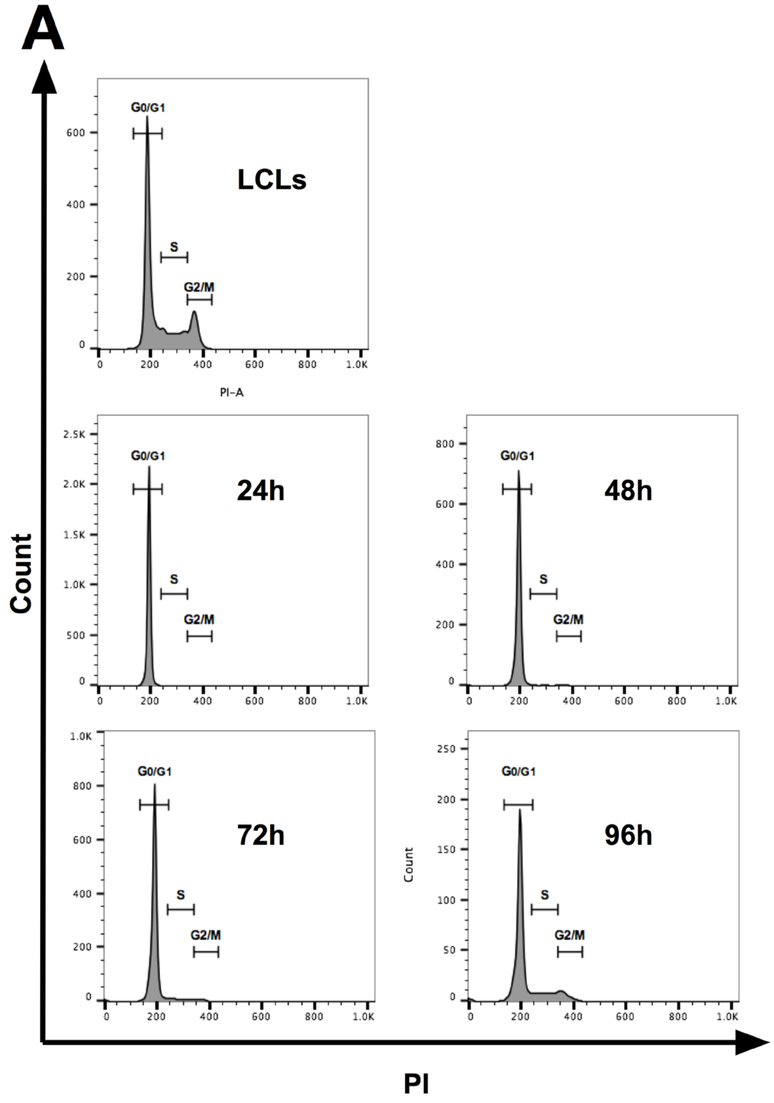


**Supplementary Figure S1: Cell cycle analysis of EBV inoculated TBCs.** (**A**) Cell populations in G1, S, G2/M at different time points post EBV-inoculation were quantified by flow cytometry based cell cycle analysis measured by labeling the fixed cells with propidium iodide (PI). LCLs were used as positive control to determine the gate for G1-S-G2/M phases. Results shown are representative from 3 independent donors.

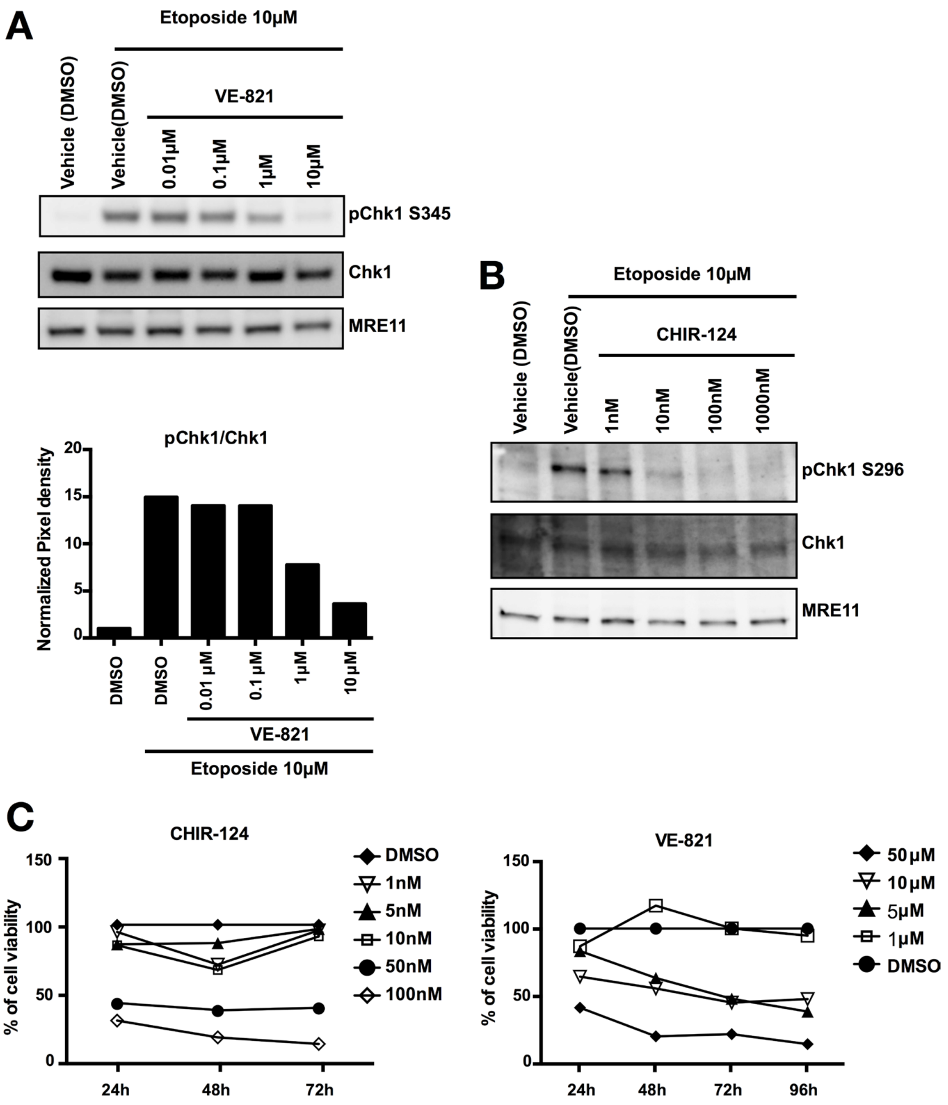


**Supplementary Figure S2: Validation of the pharmacological inhibitors VE-821 or CHIR-124.** (**A**) Inhibition of ATR was determined by measuring the expression of total Chk1 and pChk1 S345 by western blotting. Lymphoblastoid cell lines (LCLs) were treated 1 hour with Etoposide followed by a 2 hours treatment with the specific pharmacological ATR inhibitor VE-821. Chk1 phosphorylation at S345 was quantified by densitometric analysis. Data are represented as ratio of phosphorylated-to-total. Quantification was performed using the software imageJ 1.49 t. (**B**) Inhibition of Chk1 was determined by measuring the expression of total Chk1 and pChk1 S296 by western blotting. LCLs were treated 1 hour with Etoposide followed by a 2 hours treatment with the specific pharmacological Chk1 inhibitor CHIR-124. (**C**) Cell viability was measured by WST-1 assay as described in Materials and Methods. LCLs were exposed to different concentrations of VE-821 or CHIR-124 and cell viability was assessed at different time points.
